# Small RNA Sequencing Reveals Transfer RNA-derived Small RNA Expression Profiles in Retinal Neovascularization

**DOI:** 10.7150/ijms.46209

**Published:** 2020-07-06

**Authors:** Yingqian Peng, Jingling Zou, Jiang-Hui Wang, Huilan Zeng, Wei Tan, Shigeo Yoshida, Liwei Zhang, Yun Li, Yedi Zhou

**Affiliations:** 1 Department of Ophthalmology, The Second Xiangya Hospital, Central South University, Changsha, Hunan 410011, China.; 2 Hunan Clinical Research Center of Ophthalmic Disease, Changsha, Hunan 410011, China.; 3 Centre for Eye Research Australia, Royal Victorian Eye and Ear Hospital, East Melbourne, Victoria, Australia.; 4 Ophthalmology, Department of Surgery, University of Melbourne, East Melbourne, Victoria, Australia.; 5 Department of Ophthalmology, Kurume University School of Medicine, Kurume, Fukuoka 830-0011, Japan.

**Keywords:** transfer RNA-derived small RNA, small RNA sequencing, retinal neovascularization, oxygen-induced retinopathy

## Abstract

Retinal neovascularization (RNV) is characterized in retinopathy of prematurity (ROP), diabetic retinopathy (DR), and retinal vein occlusion (RVO), which leads to severe vision loss and even blindness. To reveal the altered transfer RNA-derived small RNA (tsRNA)s in RNV, and to investigate the underlying mechanisms of the altered tsRNAs involved in RNV, we carried out a small RNA sequencing to profile tsRNA expressions in the retinas of mice with oxygen-induced retinopathy (OIR) and control mice. A total of 45 tsRNAs were significantly changed (fold change ≥ 1.5 and *P* < 0.05) in the retinas of OIR mice compared with controls. Validation by quantitative real-time reverse transcription polymerase chain reaction (qRT-PCR) in four selected tsRNAs was consistent with the results of small RNA sequencing. Bioinformatics analyses identified 153 altered target genes of the four validated tsRNAs. These altered target genes were largely enriched in developmental process, cell periphery and protein binding, as well as Th1 and Th2 cell differentiation pathway. Our study suggests tsRNAs play key roles in the pathogenesis of RNV, indicating their therapeutic potential to treat patients with RNV. Moreover, small RNA sequencing is a useful tool to identify changes in tsRNA expression, an important indicator of the progress of retinal diseases.

## Introduction

Retinal neovascularization (RNV) is characterized in several kinds of retinal diseases, such as retinopathy of prematurity (ROP), diabetic retinopathy (DR), and retinal vein occlusion (RVO), which leads to severe vision loss and even blindness 1, 2. Oxygen-induced retinopathy (OIR) mouse model is a well-established and widely used animal model to study the pathogenesis of RNV and potential pharmaceutical molecules 3. OIR is induced in mice subjected to hyperoxia, a process results in abnormal development of retinal vessels and rapid growth of neovascular tufts on return to room air 4. This model mimics key pathologies in human retinal angiogenesis, such as ROP that causes blindness in infants and children and DR that causes vision impairment in adults, affecting up to 93 million of the population worldwide 5.

Although anti-vascular endothelial growth factor (VEGF) treatment has shown great benefits to patients with RNV 6, 7, there are many patients who are not responsive for this treatment. Moreover, RNV can result in several complications including vitreous hemorrhage and retinal detachment 1, which are indications of invasive surgical interventions. Besides, the great economic burden brought by anti-VEGF agents for patients and society makes it unaffordable for some patients 8-10. Thus, the limitations of current treatment modalities require new investigations to explore new targets and pathways involved in the pathogenesis of RNV.

Transfer RNAs (tRNAs) were recently found to be an important source of a group of functional non-coding RNAs, tRNA-derived small RNAs, including tRNA-derived RNA fragments (tRFs) and tRNA-derived stress-induced RNAs (tiRNAs), both of which are differentiated from each other according to the cleavage position of the mature or precursor tRNAs 11-14. An increasing body of evidence demonstrated that tsRNAs play important regulatory roles in numerous physiological and pathological processes 14, 15, such as protein synthesis 16, ribosome biogenesis 17 and cancer transcriptome 18. A number of studies have revealed that tsRNAs are implicated in human diseases, such as prostate cancer 19, 20, trastuzumab-resistant breast cancer 21, osteoporosis 22 and neurological disorders 15, 16. Induced specifically by hypoxia and mapping to the known tRNA gene, makes tRFs as a new type of potential therapeutic targets of breast cancer 23. *Li et al.* discussed the role of tsRNAs plays in ischemic pathophysiology and in post-ischemia angiogenesis. The study showed that angiogenesis could be suppressed by the tRNA^Val(CAC)^- and tRNA^Gly(GCC)^-derived small RNAs and up-regulated these fragments in ischemic tissues may alleviate angiogenesis 24. We recently reported the expression profile of several kinds of RNAs in the retinas of OIR mice, including messenger RNAs (mRNAs), long non-coding RNAs (lncRNAs), microRNAs (miRNAs) and circular RNAs (circRNAs) 25-27. However, the expression profile and role of tsRNAs in RNV remain unclear, and we hypothesized that tsRNAs may participate in retinal angiogenesis by targeting mRNAs via variable pathways. To confirm the speculation, small RNA sequencing was performed to identify the tsRNA profile in the retinas of OIR mice, and bioinformatics analyses were conducted, which might provide undiscovered mechanisms of RNA pathogenesis and develop potential therapeutic targets for retinal neovascular diseases.

## Materials and Methods

### Animals and Oxygen-induced retinopathy (OIR) mouse model

C57BL/6J mice (Hunan SJA Laboratory Animal, Changsha, China) were used in the study. Mice were treated by the rules of the ARVO Statement for the Use of Animals in Ophthalmic and Vision Research. All procedures were subjected to approval by the Institutional Animal Care and Use Committee of Central South University, China (Approval No. 2016sydw0276).

The mouse model of OIR was established as previously described 4. Briefly, newborn pups were exposed to a hyperoxia condition (75% oxygen) from postnatal day (P) 7 to P12 and then returned to the room air. The control mice were in room air continuously. Retinas were collected at P17 for up to three litters in both groups. To evaluate the success of animal modeling, another pup was randomly selected from each litter of both groups at P17, and flat-mounted retinas of these selected pups were immunofluorescent stained by fluorescein-labeled isolectin B4 (Vector Laboratories, Burlingame, CA, USA) as described 28.

### RNA isolation

Retinas from both eyes of each mouse were mixed as a sample. RNA was isolated by Trizol RNA extraction kit (Invitrogen, Carlsbad, CA, USA) followed by instructions of the manufacturer. Agarose gel electrophoresis as well as Nanodrop^TM^ instrument (Thermo Fisher Scientific, Waltham, MA, USA) were used to assess the quantity and integrity of RNA samples.

### Small RNA sequencing of tsRNAs

Pretreatment of tsRNA and library preparation, small RNA sequencing, collection and analysis of the data were conducted as described 29. In brief, each RNA sample was sequentially ligated to 3' and 5' small RNA adapters. Illumina's proprietary RT primers and amplification primers (Illumina, San Diego, CA, USA) were used to synthesize and amplify the cRNA, and PCR amplified fragments (134-160 bp) were extracted and purified from the PAGE gel. The libraries are qualified and absolutely quantified using Agilent BioAnalyzer 2100 (Agilent Technologies, Santa Clara, CA, USA). The sequencing was performed using Illumina NextSeq 500 system with a NextSeq 500/550 V2 kit (Illumina).

### Validation of tsRNAs expression level changes

Quantitative real-time reverse transcription polymerase chain reaction (qRT-PCR) was used to confirm the selected tsRNAs' sequencing results. RNA was treated by rtStar™ tRF&tiRNA Pretreatment Kit (Arraystar, Rockville, MD, USA) and then converted to cDNA using rtStar™ First-Strand cDNA Synthesis Kit (3' and 5' adaptor) (Arraystar). qRT-PCR experiments were performed on ViiA 7 Real-time PCR System (Applied Biosystems, Foster City, CA, USA) by using 2 × PCR Master Mix (Arraystar). Reaction conditions were described in our previous study 29. The 2-ΔΔCt method 30 was applied in the calculation of tsRNA expression levels normalized with U6. The primers of tested tsRNAs were listed in Table 1.

### Bioinformatics analyses

Prediction of target genes of four tsRNAs were conducted by miRanda and Targetscan. The significantly altered mRNAs with a threshold (fold change ≥ 1.5, *P* < 0.05) were selected to construct the network according to the data of our previous microarray analysis study (25). Gene Ontology (GO) (http://www.geneontology.org/) and Kyoto Encyclopedia of Genes and Genomes (KEGG) database (http://www.genome.jp/kegg/) were used for prediction of biological functions and pathways involved in RNV.

### Statistical Analyses

The small RNA sequencing data of tsRNAs was evaluated by counts per million (CPM) mapped reads. R package edgeR 31 was used for the analysis of differentially expressed RNAs. The p-values were assessed by negative binomial distribution. A threshold (fold change ≥1.5, *P* <0.05) was applied for screening significantly altered tsRNAs. The statistical difference of qRT-PCR was assessed by Student's t-test (*P* < 0.05) and showed as the mean ± SEM.

## Results

### Expression profiles of tsRNAs in the retinas of OIR and control mice

**Figure 1:**
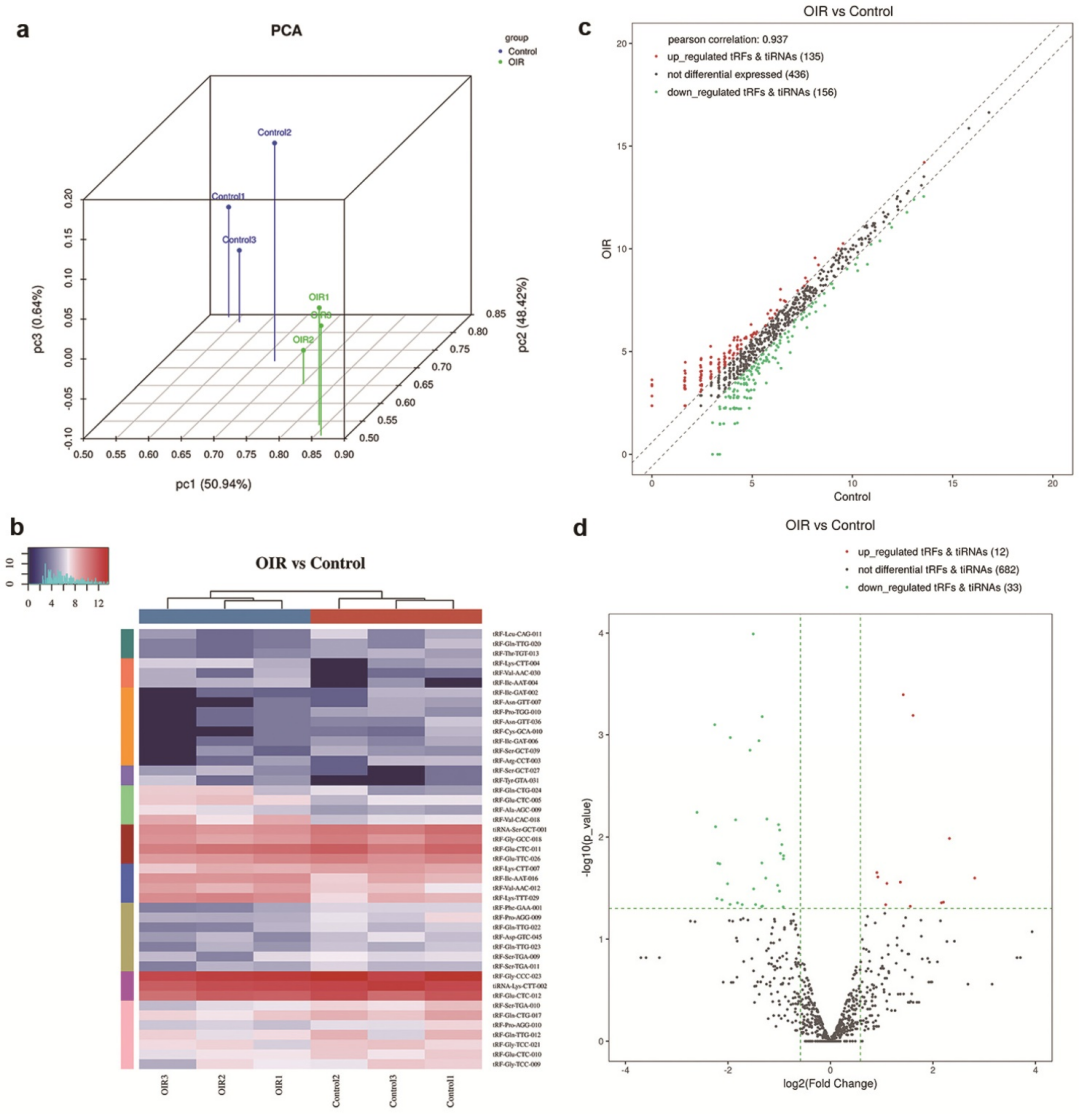
** a.** Principal Component Analysis (PCA) of tsRNAs expressions in the retinas of control mice versus OIR mice. Each point represents a sample. Blue points are the control group samples (n=3) and green points are the OIR group samples (n=3). **b.** Heatmap representation for tsRNAs of hierarchical clustering for 6 samples (3 for controls and 3 for OIR mouse model). The color in the panel represents the relative expression levels: blue and red represent low and high expression levels, respectively. **c.** The scatter plot between the retina of OIR mouse and control groups for tsRNAs. The CPM values of all tsRNAs are plotted. tsRNAs above the top line illustrated by red dots are up-regulated and those below the bottom line represented by green dots are down-regulated. Gray dots demonstrate tsRNAs that are not altered. **d.** The volcano plot of tsRNAs. Red/Green dots represent significantly up-/down-regulated tsRNAs (fold change ≥ 1.5, *P* < 0.05). Gray dots indicate tsRNAs that are non-differentially expressed.

Followed by small RNA sequencing, raw sequencing data of tsRNAs were analyzed based on alignment statistical analysis. A 3D visualization of the relationship between the samples was given by a principal component analysis (PCA) plot, which indicated a distinguishable tsRNA expression profiling among these included samples, and also suggested a clear difference between the two groups in the molecular composition (Figure 1a).

To evaluate the difference of expression level of tsRNAs in the retina of OIR and controls, a threshold of the fold change of ≥1.5 and p-value < 0.05 was applied, which resulted in significantly changed tsRNAs in a hierarchical clustering heat map (Figure 1b). The altered tsRNAs were further analyzed by a scatter plot (Figure 1c) and a volcano plot (Figure 1d). A total of 291 tsRNAs were altered in OIR mice compared to controls. Among these, 135 tsRNAs were up-regulated while 156 tsRNAs were down-regulated in the retinas of OIR mice (Figure 1c). Moreover, 45 altered tsRNAs were further identified in OIR mice compared to controls when a threshold fold-change ≥1.5 and P<0.05 was applied. Of these, 12 tsRNAs were markedly up-regulated, and 33 were down-regulated in the retina of OIR mice (Figure 1d). Among these 45 altered tsRNAs, tRF-Tyr-GTA-031 was the most up-regulated tsRNA and tRF-Ile-GAT-002 was the most down-regulated tsRNA in OIR retinas (Table 2).

### tsRNAs expression catalog of OIR and control mice

**Figure 2:**
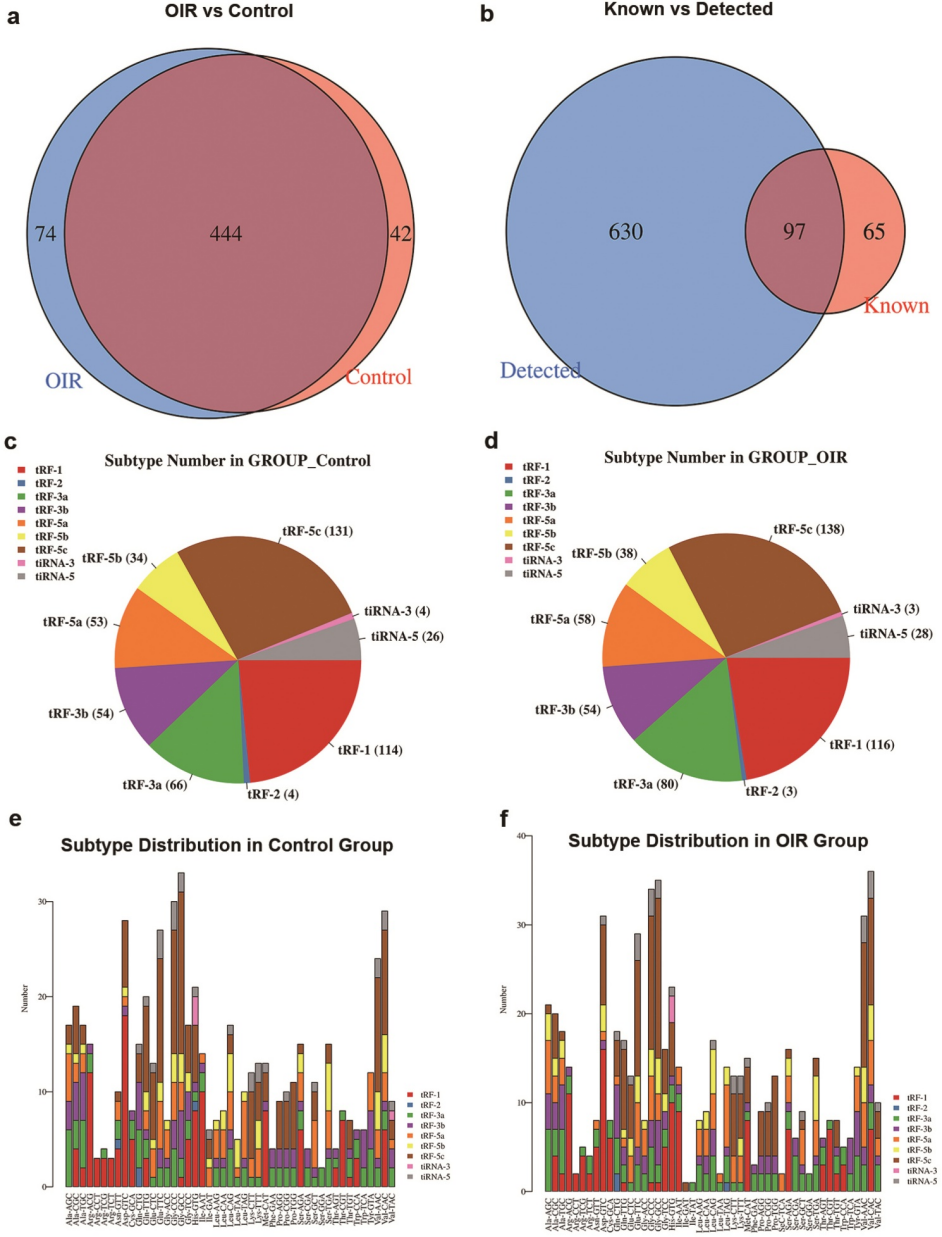
** a.** Venn diagram of commonly and specifically expressed tsRNA numbers in control and OIR mice. **b.** Venn diagram of the numbers of tsRNA known (according to tRFdb) and detected from the small RNA sequencing. **c.** and **d.** Pie chart of subtype tsRNAs in control (**c**) and OIR mice (**d**). The values represented the number of subtype tsRNAs. The color represents the subtype tsRNAs (CPM ≥ 20). **e. and f.** The number of tsRNAs in subtypes against tRNA isodecoders in control (**e**) and OIR mice (**f**)**.** The X axes represent tRNA isodecoders and the Y axes show the number of all subtype tsRNAs against tRNA isodecoders in two groups. The color represents the subtype tsRNAs (CPM ≥ 20).

A Venn diagram was plotted with R VennDiagram package to investigate the shared and specifically expressed tsRNAs between the retina of OIR and controls. The results showed that there were 444 tsRNAs shared between both groups, while 74 and 42 tsRNAs were uniquely expressed in OIR and controls, respectively (Figure 2a). There were 97 identified tsRNAs were known from tRFdb (32, 33) (Figure 2b). To examine whether tsRNA expression was changed in the retinas of OIR mice, the numbers of tsRNA transcripts subtype were estimated in both groups. Pie charts illustrated the RNA number in each subtype (with the average CPM ≥20). In the controls, 131 tRF-5c, 34 tRF-5b, 53 tRF-5a, 54 tRF-3b, 66 tRF-3a, 4 tRF-2, 114 tRF-1, 26 tiRNA-5 and 4 tiRNA-3 were identified (Figure 2c). However, 138 tRF-5c, 38 tRF-5b, 58 tRF-5a, 54 tRF-3b, 80 tRF-3a, 3 tRF-2, 116 tRF-1, 28 tiRNA-5 and 3 tiRNA-3 were identified in OIR retinas (Figure 2d). The distribution of the numbers of tsRNA transcripts is different except for tRF-3b between groups. In addition, the stacked chart demonstrated the numbers of tsRNAs derived from the variable anticodon tRNAs in both control and OIR groups (Figure 2e, f), from which the subtype distribution in these two groups is considered to be totally different.

### Validation of sequencing results by qRT-PCR

**Figure 3:**
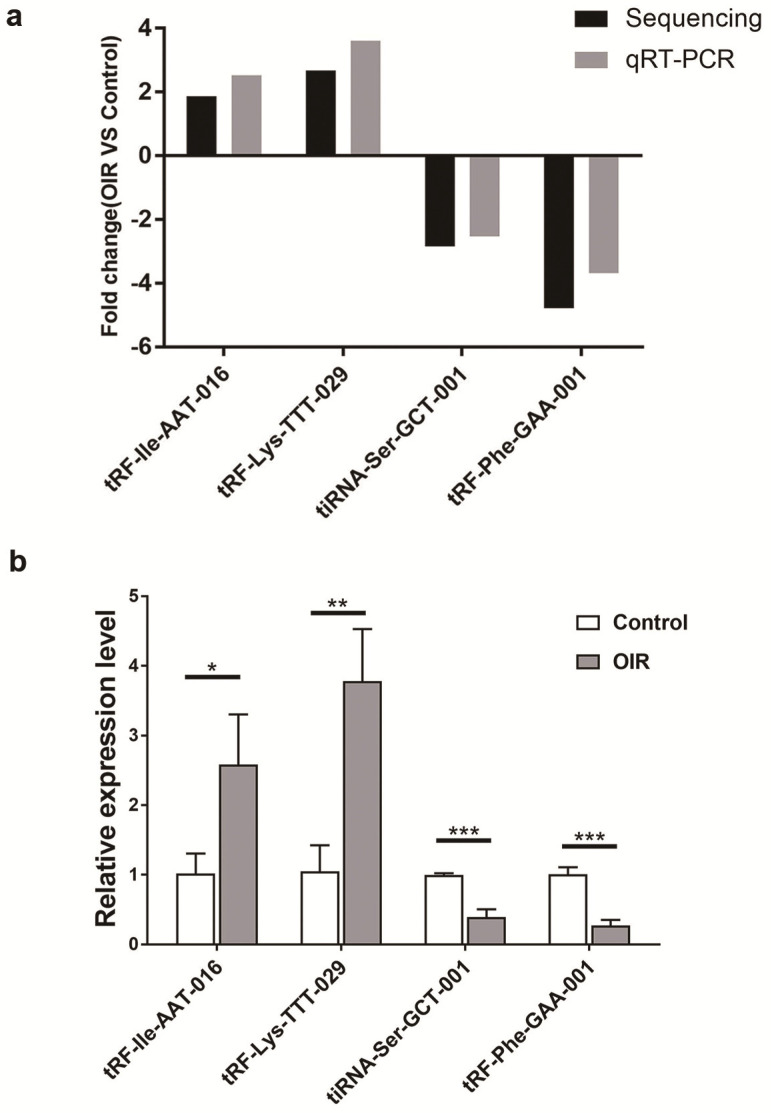
** Validation by qRT-PCR in OIR and control mice. a.** Fold change of qRT-PCR analysis confirmed tsRNA expression changes of RNA-seq-identified tsRNAs in the retina of OIR and control mice. Four significantly altered tsRNAs were validated. **b.** Relative expression levels of these tsRNAs were assessed by qRT-PCR. tRF-lle-AAT-016, tRF-Lys-TTT-029, tiRNA-Ser-GCT-001 and tRF-Phe-GAA-001 are showed to be statistically different between these two groups. Each bar represents the relative expression level of tsRNA tested in control vs OIR. *, *P* < 0.05; **, *P* < 0.01; ***, *P* <0.001.

qRT-PCR analysis of altered tsRNAs in OIR and control mice was carried out to confirm the results obtained by the small RNA sequencing. Four altered tsRNAs (tRF-lle-AAT-016, tRF-Lys-TTT-029, tiRNA-Ser-GCT-001 and tRF-Phe-GAA-001) were randomly selected. qRT-PCR results showed a consistent trend with small RNA sequencing results (Figure 3a). The expression level of tRF-lle-AAT-016 and tRF-Lys-TTT-029 were significantly up-regulated, while the expression levels of tiRNA-Ser-GCT-001 and tRF-Phe-GAA-001 were down-regulated in OIR compared to controls (Figure 3b).

### Target gene prediction by bioinformatics analysis

**Figure 4:**
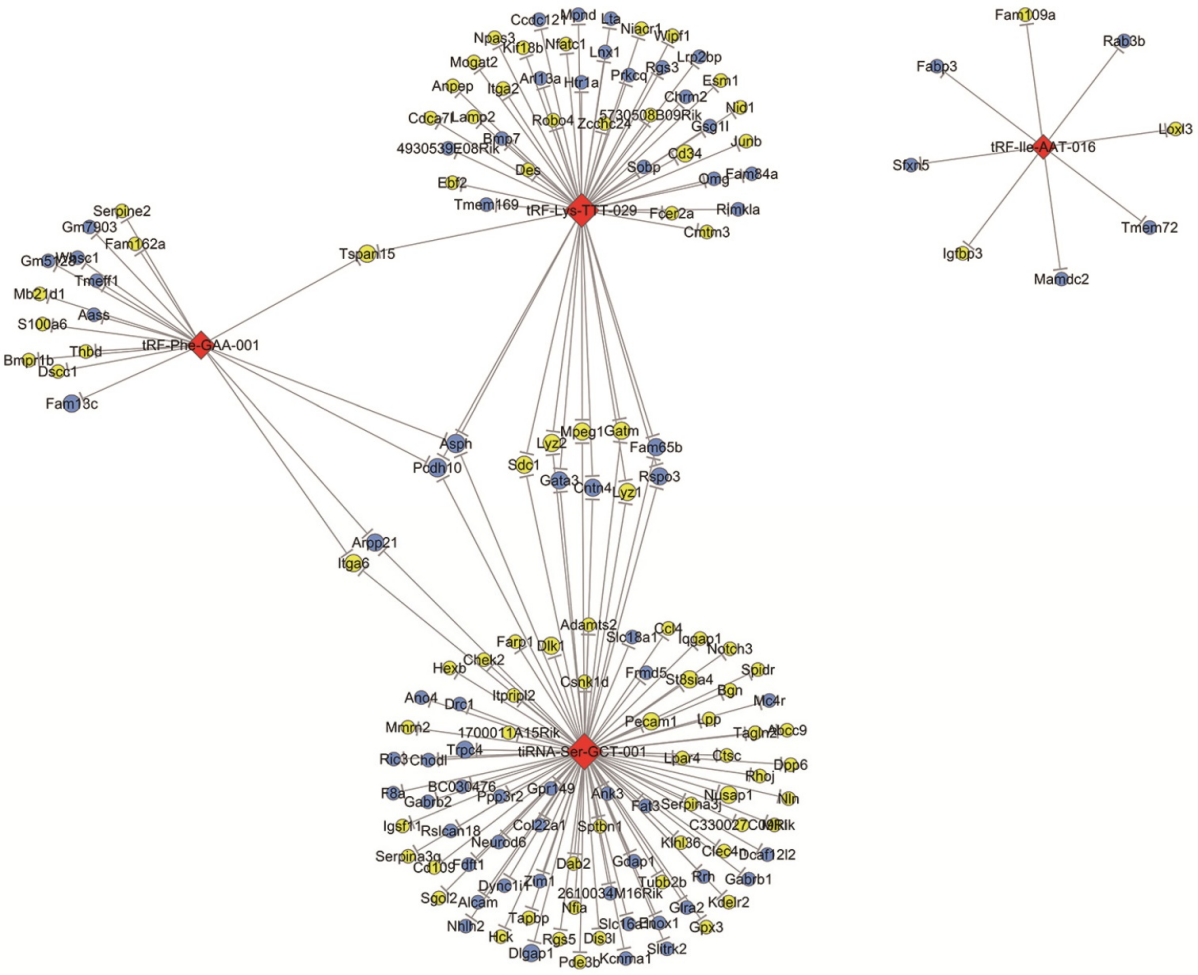
** The network of validated tsRNAs and potential altered target mRNAs.** Yellow ellipse nodes represent up-regulated mRNAs, blue ellipse nodes represent down-regulated mRNAs, and red diamond nodes represent validated tsRNAs. All results are with a threshold ≥1.5-fold change.

According to the tsRNA-target gene network based on TargetScan and miRanda, we identified that there were 2252 target genes associated with four validated tsRNAs. These target genes were intersected with significantly changed mRNAs with a threshold of ≥1.5-fold change and a p-value of <0.05 in OIR mice according to a microarray analysis in our previous study 25. The network of tsRNA-mRNA revealed that 153 mRNAs were altered and targeted by four tsRNAs (tRF-lle-AAT-016, tRF-Lys-TTT-029, tiRNA-Ser-GCT-001 and tRF-Phe-GAA-001) (Figure 4). Specifically, both tRF-Phe-GAA-001 and tRF-Lys-TTT-029 regulate Tspan15, and both tRF-Phe-GAA-001 and tiRNA-Ser-GCT-001 regulates four mRNAs (Podh10, Asph, Itga6 and Arpp21). tiRNA-Ser-GCT-001 and tRF-Lys-TTT-029 are connected by 11 shared targeted genes including Podh10, Asph, Sdc1, Lyz2, Mpeg1, Gatm, Fam65b, Rspo3, Gata3, Cntn4 and Lyz1. Interestingly, tRF-Phe-GAA-001, tRF-Lys-TTT-029 and tiRNA-Ser-GCT-001 are linked by two down-regulated mRNAs including Podh10 and Asph.

### GO and KEGG analyses

**Figure 5:**
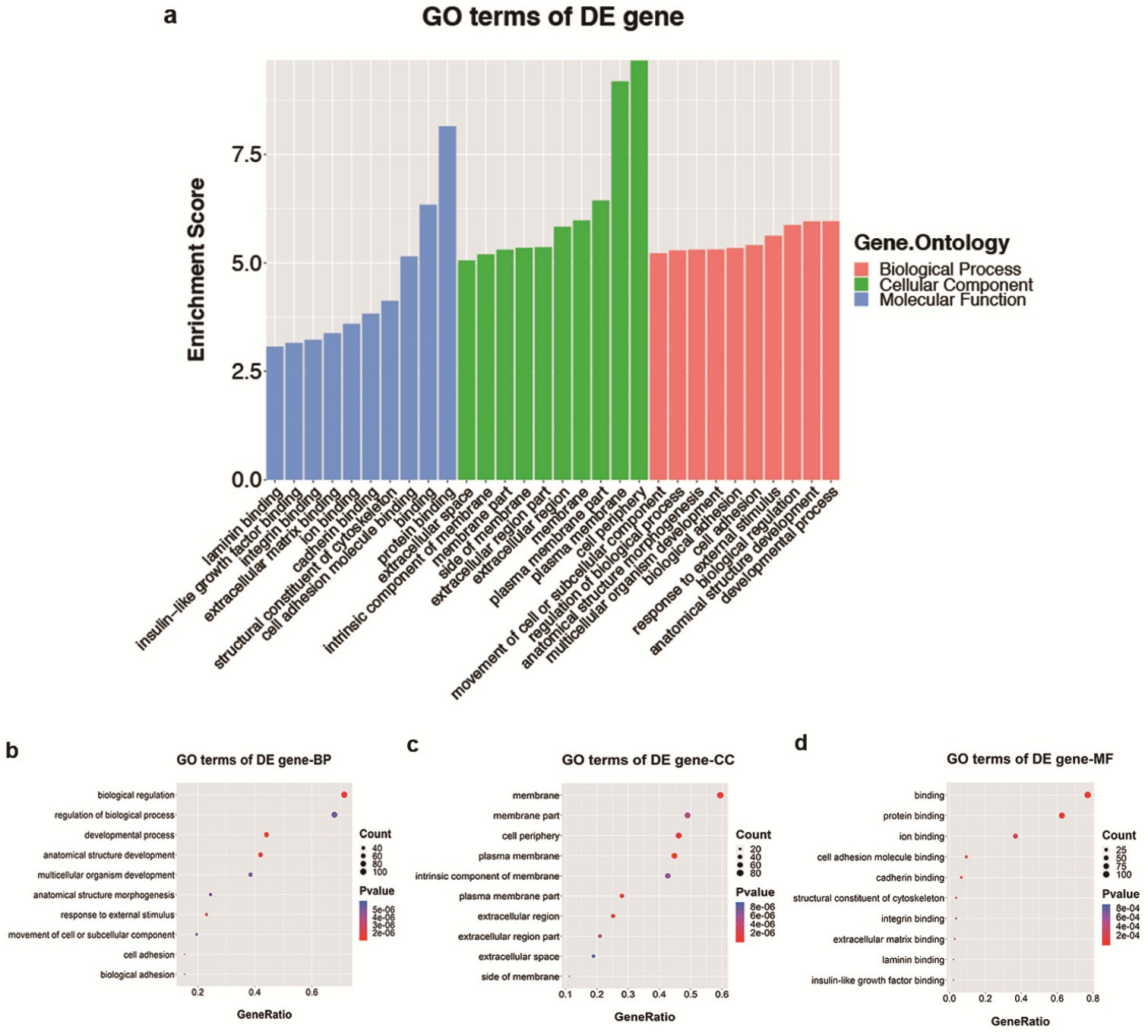
** The GO analysis of altered target mRNAs of the validated tsRNAs. a.** Bar plot explanation (enrichment score): top 10 significant enrichment terms in three domains. **b-d.** Dot plot explanation (gene ratio): gene ratio values of the top 10 most significant enrichment terms in biological process (**b**), cellular component (**c**) and molecular function (**d**). The most reliable one considered to be the one with the largest gene counts and lowest p-value.

**Figure 6:**
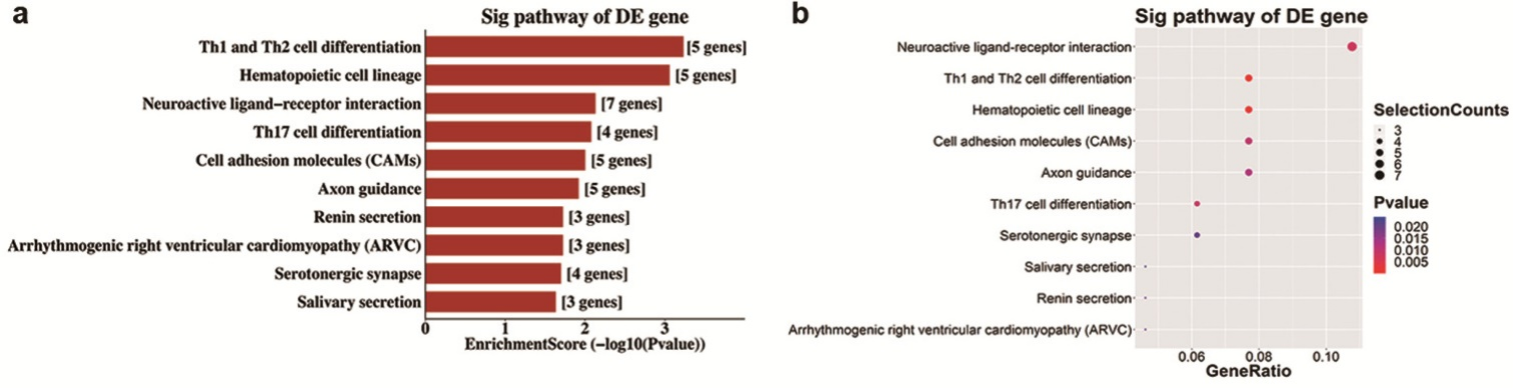
** KEGG pathway analysis of altered target mRNAs of the validated tsRNAs. a.** Pathway barplot explanation (enrichment score): top 10 significant enrichment pathways. **b.** Pathway dotplot explanation (gene ratio): gene ratio value of the top 10 most significant enrichment pathways.

Analyses of GO and KEGG pathway analyses were performed according to the predicted target genes with a threshold of ≥1.5-fold change and a p-value of <0.05 to further explore the possible involved functions and pathways of these altered tsRNAs. The most enriched oncology in three included domains (biological process, cellular component and molecular function) are developmental process (GO: 00032502), cell periphery (GO: 0071944) and protein binding (GO: 0005515) (Figure 5a), respectively. The dot plot revealed the gene ratio values of the top ten most significant enrichment terms in these three domains (Figure 5b-d). As the plots shown in biological process, the most reliable one is developmental process; and in cellular component, it is considered to be located in the cell periphery; and in molecular function, the most reliable one is protein binding. Moreover, KEGG pathway analysis revealed that Th1 and Th2 cell differentiation pathway is involved in the regulation of target genes of altered tsRNAs (Figure 6a and b). There are five genes involved in this pathway, including GATA3, NFATC1, NOTCH3, PPP3R2 and PRKCQ (Table 3).

## Discussion

Although increasing evidence shows that miRNAs and tsRNAs play crucial roles in choroidal neovascularization 29, 34, few studies investigated how does tsRNA involve in RNV pathogenesis. In this study, we carried out a small RNA sequencing of tRFs and tiRNAs to profile their expression in the retinas of OIR mice.

Similar to miRNAs, tsRNAs are able to regulate gene expressions at post-transcriptional level through binding corresponding sequencing in mRNAs 35. An increasing number of investigations revealed significant regulatory roles of tsRNAs in both physiological and pathological processes 15, 36. tsRNA contributes to many pathological processes including stress and viral infection, cancer, neurodegeneration, metabolic syndromes and microbiome dysregulation 37. A breast cancer study investigating hypoxia-induced chemoresistance showed that tRFs were induced specifically by hypoxia and mapped to the known tRNA gene, suggesting it plays regulatory roles and could be potential therapeutic targets 23. In addition, tsRNA in plasma exosomes could serve as a novel diagnostic biomarker for cancer diagnosis 38.

Our small RNA sequencing data suggested that there were significant differences in retinal tsRNA expression between OIR mice and controls. GO analysis implied that target genes of altered tsRNAs involved in developmental process, indicating that tsRNAs may also play some roles in developmental process of RNV. tsRNAs might also be involved in protein binding, implying tsRNA could be a mediator to regulate protein interaction and contribute to RNV formation, which is similar to the mechanisms of cancer studies 18, 39, 40.

Network analysis identified that Tspan15 is the only up-regulated gene targeted by tRF-Phe-GAA-001 and tRF-Lys-TTT-029, both of which were up-regulated in OIR mice. A study in hepatocellular carcinoma reported that cell proliferation is significantly increased by Tspan15 overexpression in HepG2 cell line led to cell proliferation by regulating abundant membrane proteins to promote expression of growth factor receptors 41.

The KEGG pathway analysis demonstrated that Th1 and Th2 cell differentiation pathway is the most enriched and reliable pathway involving altered target genes of four validated altered tsRNAs. As we reported in an earlier study, M2 macrophages increased a larger extent than M1 macrophages in the retinas of OIR mice 41, and a typical Th1 cytokine, IL-12, was significantly decreased in OIR retinas 42. Moreover, Th1 and Th2 cytokines have different functions in ocular neovascularization 43. Thus, Th1 and Th2 cell differentiation pathway could be potentially important in the pathogenesis of RNV, which is worth to be further investigated.

As we discussed before, tRFs can be induced specifically by hypoxia, making it a novel type of regulatory factors as well as therapeutic targets 23. In OIR mice, tsRNAs expressed in the retina may be involved in RNV formation possibly by specific cleavage in the anticodon loops of mature tRNAs, and affecting regulated gene expression, which has been indicated in other diseases 15.

In this study, we found a total of 45 tsRNAs were significantly altered in OIR mice compared to controls, which demonstrated a dysregulation of tsRNA in OIR mouse model and RNV related diseases. In addition, through bioinformatics analyses, we identified numerous targeted genes that are related with four validated altered tsRNAs. Furthermore, by GO and KEGG analyses, we detected several potential biological functions and possible involved pathways that might contribute to RNV pathogenesis, which also provided potential novel targets for therapies of retinal neovascular diseases.

There are several limitations in the study. First of all, the expression profiles of the tsRNAs in variable period of OIR pathogenesis have not been examined. It has been shown that neovascular regression occurs from P17 to P25 in OIR mouse model 4, some of the altered tsRNAs might be normalized during the later stage of this model. The potential change at the later stage of RNV pathogenesis remained unknown which is worth to be revealed. In addition, although the OIR mouse model by exposure of 75% oxygen is widely used for investigation of RNV, it cannot accurately reflect the retinal neovascular diseases in human, and the vascular damage is different. For instance, vascular obliteration of the central retinal vessels exists in OIR mice, while ROP mainly affect the development of peripheral retinal vessels in human patients 44. Therefore, it is necessary to confirm the findings of the study in the samples of human ROP patients in the future. Moreover, further investigations are also needed to explore the functions and mechanisms of specific tsRNAs in patients with retinal neovascular diseases.

In summary, we identified altered tsRNAs in the retinas of OIR mice and predicted potential target genes which might be involved in RNV pathogenesis. All these preliminary data revealed that tsRNAs could affect and participate in the pathological processes in OIR mouse model and RNV formation, suggesting that tsRNAs could serve as potential biomarkers to identify retinal neovascular diseases, as well as novel therapeutic targets for patients with retinal neovascular diseases, an alternative to anti-VEGF therapy.

## Figures and Tables

**Table 1:** Sequence of primers used for qRT-PCR

Gene name	Primer sequence	Tm (°C)	Product length (bp)
U6	F:5'GCTTCGGCAGCACATATACTAAAAT 3' R:5'CGCTTCACGAATTTGCGTGTCAT 3'	60	89
tiRNA-Ser-GCT-001	F:5'CCGACGATCAAGAAAGATTGC 3' R:5' TGTGCTCTTCCGATCTCAGTTC 3'	60	45
tRF-Ile-AAT-016	F:5'TCCGACGATCTCCCCGTAC 3' R:5' TGCTCTTCCGATCTTGGTGG 3'	60	42
tRF-Lys-TTT-029	F:5' CGACGATCTCCCTGTTCGG 3' R:5' TGCTCTTCCGATCTTGGCG 3'	60	39
tRF-Phe-GAA-001	F:5' AGTCCGACGATCAATTGTATCC 3' R:5' GCTCTTCCGATCTTGGTGTTTAT 3'	60	46

**Table 2:** Top 10 up- and down-regulated tsRNAs in OIR mouse model

tRF_ID	Type	Length	Regulation	Fold change	p-Value
tRF-Tyr-GTA-031	tRF-5b	23	up	7.031566	0.025213
tRF-Ile-AAT-004	tRF-5a	14	up	4.998730	0.010319
tRF-Ser-GCT-027	tRF-3a	17	up	4.606634	0.043493
tRF-Val-AAC-030	tRF-5c	31	up	4.482010	0.044018
tRF-Val-CAC-018	tRF-3c	32	up	3.055908	0.000645
tRF-Lys-CTT-004	tRF-5a	16	up	2.943952	0.047675
tRF-Lys-TTT-029	tRF-3a	17	up	2.676338	0.000403
tRF-Ala-AGC-009	tRF-3a	17	up	2.573722	0.027642
tRF-Glu-CTC-005	tRF-5a	15	up	2.143898	0.028453
tRF-Gln-CTG-024	tRF-2	14	up	2.109806	0.046034
tRF-Ile-GAT-002	tRF-5a	15	down	-6.077296	0.005728
tRF-Phe-GAA-001	tRF-3b	21	down	-4.781254	0.000797
tRF-Leu-CAG-011	tRF-5c	31	down	-4.727424	0.007916
tRF-Asn-GTT-007	tRF-2	15	down	-4.639328	0.040046
tRF-Pro-TGG-010	tRF-5c	29	down	-4.587210	0.018052
tRF-Asn-GTT-036	tRF-5c	31	down	-4.465570	0.0182937
tRF-Cys-GCA-010	tRF-3b	19	down	-4.336246	0.041290
tRF-Ile-GAT-006	tRF-5b	24	down	-4.019039	0.028686
tRF-Gln-TTG-020	tRF-5b	23	down	-3.885536	0.045692
tRF-Pro-AGG-009	tRF-5c	28	down	-3.875247	0.001064

**Table 3:** Top 10 KEGG pathways of differentially expressed target mRNAs based on validated tsRNAs in OIR vs. control

PathwayID	Definition	Selection Counts	Fisher-P value	Genes
mmu04658	Th1 and Th2 cell differentiation - Mus musculus (mouse)	5	0.00059063	GATA3//NFATC1//NOTCH3//PPP3R2//PRKCQ
mmu04640	Hematopoietic cell lineage - Mus musculus (mouse)	5	0.00747224	ANPEP//CD34//FCER2A//ITGA2//ITGA6
mmu04080	Neuroactive ligand-receptor interaction - Mus musculus (mouse)	7	0.00845279	CHRM2//GABRB1//GABRB2//GLRA2//HTR1A//LPAR4//MC4R
mmu04659	Th17 cell differentiation - Mus musculus (mouse)	4	0.01005266	GATA3//NFATC1//PPP3R2//PRKCQ
mmu04514	Cell adhesion molecules (CAMs) - Mus musculus (mouse)	5		ALCAM//CD34//ITGA6//PECAM1//SDC1
mmu04360	Axon guidance - Mus musculus (mouse)	5	0.01214082	BMP7//BMPR1B//PPP3R2//RGS3//TRPC4
mmu04924	Renin secretion - Mus musculus (mouse)	3	0.01905765	KCNMA1//PDE3B//PPP3R2
mmu05412	Arrhythmogenic right ventricular cardiomyopathy (ARVC) - Mus musculus (mouse)	3	0.01905765	DES//ITGA2//ITGA6
mmu04726	Serotonergic synapse - Mus musculus (mouse)	4	0.02017393	GABRB1//GABRB2//HTR1A//SLC18A1
mmu04970	Salivary secretion - Mus musculus (mouse)	3	0.02351413	KCNMA1//LYZ1//LYZ2
